# Influence of education and subjective financial status on dietary habits among young, middle-aged, and older adults in Japan: a cross-sectional study

**DOI:** 10.1186/s12889-023-16131-7

**Published:** 2023-06-26

**Authors:** Maki Nishinakagawa, Ryota Sakurai, Yuta Nemoto, Hiroko Matsunaga, Toru Takebayashi, Yoshinori Fujiwara

**Affiliations:** 1 Research Team for Social Participation and Community Health, Tokyo Metropolitan Institute for Geriatrics and Gerontology, 35-2 Sakae-cho, Itabashi-ku, Tokyo, 173-0015 Japan; 2 Department of Preventive Medicine and Public Health, Keio University School of Medicine, 35 Shinanomachi, Shinjuku-ku, Tokyo, 160-8582 Japan

**Keywords:** Socioeconomic status, Education, Subjective financial status, Dietary habits, Breakfast, Balanced meals

## Abstract

**Background:**

Research has suggested an association between lower socioeconomic status (SES) and unhealthy dietary habits. However, differences in the effects of different SES indicators and age remain unclear. The current study addressed this research gap by investigating the relationship between SES and unhealthy dietary habits, specifically focusing on educational attainment and subjective financial status (SFS) among varied age groups.

**Methods:**

Data were derived from a mail survey of 8,464 people living in a suburb of Tokyo, Japan. Participants were classified into three age groups (20–39 years: young adults; 40–64 years: middle-aged adults; and 65–97 years: older adults). SES was assessed based on individual educational attainment and SFS. Unhealthy dietary habits were defined as skipping breakfast and a low frequency of balanced meal consumption. Participants were asked how often they ate breakfast, and those who did not respond “every day” were categorized as “breakfast skippers.” Low frequency of balanced meal consumption was defined as eating a meal that included a staple meal, main dish, and side dishes at least twice a day for less than five days per week. Poisson regression analyses with robust variance adjusted for potential covariates were used to determine the interactive effects of educational attainment and SFS on unhealthy dietary habits.

**Results:**

Individuals with lower educational attainment across all age groups skipped breakfast more frequently compared to those with higher educational attainment. For older adults, poor SFS was associated with skipping breakfast. Young adults with poor SFS and middle-aged adults with lower educational attainment tended to eat less balanced meals. In addition, an interaction effect was found in older adults, where those with lower education despite good SFS and those with poor SFS despite higher education were at a greater risk of falling into unhealthy diet.

**Conclusions:**

The findings suggested that different SES indicators affect healthy dietary habits in different generations, and therefore, health policies should consider the potential influence of different SES on promoting healthier dietary habits.

**Supplementary Information:**

The online version contains supplementary material available at 10.1186/s12889-023-16131-7.

## Introduction

A balanced meal that includes a wide variety of foods and provides adequate nutrients and energy is important for maintaining and promoting good health [1]. An unhealthy diet is a major risk factor for malnutrition and morbidity in non-communicable chronic diseases, resulting in increased mortality [2]. As a result, many countries have implemented public health policies that recommend a balanced meal based on a wide variety of foods as a public health policy. In Japan, a common meal style that includes a combination of a staple food, main dish, and side dish is considered a balanced meal. Previous findings suggest that consuming such meals at least twice a day may ensure adequate intake of many food groups and nutrients [3, 4]. Therefore, eating balanced meals is an important dietary habit for maintaining good health.

In addition, breakfast consumption is crucial component of a healthy dietary habit [5]. Skipping breakfast can lead to poor dietary quality, which increases the risk of obesity and chronic diseases [6–9]. Furthermore, skipping breakfast is associated with unhealthy behaviors, including smoking, excessive alcohol consumption, and a sedentary lifestyle in both adolescents and adults [10]. These findings suggest that reducing the number of people who skip breakfast is an effective health measure for promoting good health.

In Japan, a low frequency of balanced meal consumption and skipping breakfast are both recognized as important public health issues under the national health promotion program, named “Health Japan 21” [11, 12]. Examining the factors contributing to these unhealthy dietary habits can help identify barriers to adequate nutrition and healthy lifestyles, leading to the early detection of people who are at high risk of experiencing adverse health outcomes. Individual socioeconomic status (SES) influences such dietary habits [13, 14]. For instance, low education and income have been associated with unhealthy dietary habits, including consuming less balanced meals and skipping breakfast [10, 14]. By contrast, higher educational attainment or occupational position (an indicator of higher income) is likely to result in the maintenance of a healthy diet [15]. These findings are reasonable because individuals with lower educational attainment may lack knowledge of healthy dietary behaviors [15], and those with lower income may struggle to afford a variety of foods [16].

Studies have suggested a close relationship between SES and unhealthy dietary behaviors. One study found that an unhealthy diet associated with lifestyle-related factors was consistently observed across both low and high educational levels [17]. This finding indicates that education may not always affect dietary behaviors. Considering the definition of SES, it is possible that income level, an indicator of SES, could confound these results because actual financial status directly influences to the affordability of higher-quality diets. In particular, income level may interact with educational level, thereby modulating the association between education and dietary behaviors. These assumptions are partly supported by the finding that subjective financial status (SFS) has been associated with a decline in mental and self-rated health, independent of educational level [18]. Therefore, the well-known association between education and dietary behaviors may be modulated by another aspect of SES, which is SFS. However, few reports have investigated the interactive effect of education and SFS on dietary behaviors.

Therefore, this study aimed to examine the interactive effects of education and income levels on dietary habits, with assessments of the frequency of balanced meal and breakfast consumption as the main outcomes. Based on previous findings indicating that an unhealthy diet was consistently observed at both lower and higher educational levels, we hypothesized that the associations between education and dietary habits would differ according to income level and vice versa. To this end, the current study analyzed cross-sectional data from a mail survey focusing on three generations (young, middle-aged, and older adults) to examine age-related differences, which have rarely been explored in existing research. Understanding this interaction between SES and generation on dietary habits could provide valuable insights into the factors influencing unhealthy dietary behaviors.

## Methods

### Study design and participants

Data were extracted from a mail survey conducted in 2019 in a town located in a suburb of Tokyo, Japan with a population of 259,573, of which the working-age population aged between 15 and 64 years and the population aged 65 years or older comprised 65.1% and 21.7% of the total population, respectively, as of April 2019. The design and logistics of the survey have been described in detail elsewhere [19]. The survey aimed to assess the health policy of the municipality and was conducted among 21,300 individuals (9,167 young adults aged 18–39 years, 6,772 middle-aged adults aged 40–64 years, and 5,361 older adults aged 65 years or older) who were randomly selected from residents aged 18 years and older based on the basic resident register. People of foreign nationality, institutionalized older adults, and those registered as needing Care Level 4 in the national long-term care insurance system (i.e., those with activities of daily living [ADL] disabilities) were excluded from the survey. We also excluded respondents with missing values for the SES and dietary questionnaires and individuals under the age of 20 because they are legally prohibited from smoking and drinking.

This study was conducted in accordance with the ethical standards of the Declaration of Helsinki. All study procedures were approved by the Ethics Committee of the Tokyo Metropolitan Institute for Geriatrics and Gerontology (No. 2019-16).

### Measurements

#### Educational attainment and subjective financial status

To assess educational attainment, participants were asked, “ How many years in total have you attended school?”. The following four response choices were provided: (1) < *10 years (graduate of junior high degree)*, (2) *10–12 years (high school graduation degree)*, (3) ≥ 13 years (junior college, college/university, or graduate degree, including while in college or university), (4) *I don’t want to answer*. Participants who answered (4) were excluded from the analysis. Based on previous studies, the remaining participants were categorized into two groups, “lower educational attainment” (< 13 years: graduate of junior high/high school) and “higher educational attainment” (≥ 13 years: junior college, college/university, or graduate degree, including while in college or university) [20]. We confirmed that the years of education among participants was at least nine years, and they all graduated from junior high school.

SFS was used as a measure of socioeconomic status. SFS is an independent socioeconomic predictor, which is defined as an individual’s perception of their position in the social hierarchy [21]. It is strongly associated with health outcomes, especially among older adults [19, 22]. In this study, SFS was determined based on the question, “Generally speaking, do you have financial leeway?” Participants were asked to respond with one of the following: (1) *very well off*, (2) *fairly well off*, (3) *average*, (4) *tightly financed*, or (5) *very tightly financed*. The first three responses were categorized as moderate SFS, while the last two as poor SFS [19, 23].

#### Low frequency of balanced meal consumption and skipping breakfast

In this study, two different measures of unhealthy dietary habits, low frequency of balanced meal consumption and skipping breakfast, were used as independent variables. To assess the frequency of balanced meal consumption, the following question from a national survey [24] was used: “How many days a week do you eat at least two meals a day that combine a staple meal, main dish, and side dish?”, with four possible responses: (1) *almost every day*, (2) *4 or 5 days a week*, (3) *2 or 3 days a week*, (4) *rarely*. Participants who answered anything other than (1) to this question were defined as low frequency of balanced meal consumption [12]. Regarding skipping breakfast, participants were asked to answer the following question: “How often do you eat breakfast? Every day, 5 or 6 days a week, 3 or 4 days a week, 1 or 2 days a week, and rarely.” Those who did not respond with “every day” were classified as “breakfast skippers”. This definition of skipping breakfast is concordant with previous research, suggesting the association of skipping breakfast with health-compromising behaviors and an increased risk of adverse health outcomes [7, 8, 10].

#### Covariates

Relevant sociodemographic and clinical variables, including sex, age, living arrangement, marital status, current work status, comorbidities, mental distress, and instrumental ADL (IADL) were assessed as covariates to explore associations between educational attainment and SFS and dietary habits. Comorbidities were assessed for hypertension, diabetes, stroke, heart disease, and cancer. Distress over a period of four weeks was measured using the 6-item Kessler Psychological Distress Scale (K6) [25], which has a maximum score of 24, with higher scores indicating more severe depressive symptoms. IADL levels were evaluated only in the older participants (≥ 65 years) using the Tokyo Metropolitan Institute of Gerontology Index of Competence [26].

### Statistical analysis

To assess age-related differences in the associations between education, SFS, and unhealthy dietary habits, stratified analyses were performed by age group: young adults aged 20–39 years, middle-aged adults aged 40–64 years, and older adults aged 65 years and older.

A Poisson regression model with robust variance adjusted for potential covariates was employed to determine the interactive effects of educational attainment and SFS on a low frequency of balanced meal consumption and skipping breakfast separately. Educational attainment, SFS, and their interactions were included as independent variables. When a significant interaction was confirmed, we performed a simple main effect test to investigate the nature of the interaction between groups regarding educational attainment and SFS. This test helped determine whether effect of educational attainment on unhealthy dietary habits depended on the degree of SFS (moderate or poor), or conversely, whether the effect of SFS depended on educational level (higher or lower).

The percentage of participants for whom covariates were missing across each variable ranged from 0 to 3.3%, 0 to 2.5%, and 0 to 16.9% for young, middle-aged, and older adults, respectively. For the entire analysis model, the percentages of those with one or more missing values were 5.3%, 7.1%, and 28.4% for young, middle-aged, and older adults, respectively. The missing values were supplemented using multiple imputation methods, which were performed using chained equations under the assumption that the missing mechanism was missing at random. Logistic regression analysis was performed to supplement the missing data, with the missing variable as the objective variable and the non-missing variable as the explanatory variable. Regression models were adjusted for sex, age, living arrangement, marital status, current work status, comorbidity, K6, and IADL (for older adults only). The analytical model was fitted to each of the 50 datasets, and odds ratios (OR) and 95% confidence intervals (CI) were calculated and integrated based on Rubin’s multiple imputation method [27]. To evaluate the sensitivity of the theoretical hypotheses on the mechanism of missing data, the same analytical model used for the complemented data was applied to the complete-case analysis.

Because our statistical methodology permitted the repetition of the regression model twice in each age group, it increased the risk of type 1 error. Accordingly, a Bonferroni correction of *p* < 0.025 (0.05/2) for the Poisson regression models with robust variance and *p* < 0.0125 (0.05/4) for an additional simple main effect test was applied. All statistical analyses were performed using IBM SPSS Statistics version 25.0 (IBM Corporation, Armonk, NY, USA).

## Results

Of the 21,300 questionnaires sent, 9,250 were returned, resulting in a response rate of 43.4%, of which 49 responses consisted of uncertain data (e.g., completely invalid data). In addition, we excluded individuals aged < 20 years (n = 281) and those who did not answer their SES and dietary information (n = 175). Finally, a total of 8,464 participants, including 2,185 young adults (mean age 31.2 ± 5.6 years, 61.4% women), 3,008 middle-aged adults (mean age 52.0 ± 7.1 years, 55.6% women), and 3,271 older adults (mean age 75.0 ± 6.6 years, 54.6% women) were included in the analysis. Table 1 shows the sociodemographic characteristics of participants by age group.

**Table 1:** Distributions of study participant characteristics stratified by age group

Variables	Young adults (n = 2,185)	Middle-aged adults (n = 3,008)	Older adults (n = 3,271)
Sex, male	38.6%	44.4%	45.4%
Age (years), mean (SD)	31.2 (5.6)	52.0 (7.1)	75.0 (6.6)
Low frequency of BM consumption	62.9%	51.6%	24.2%
Skipping breakfast	43.0%	28.3%	14.8%
Subjective financial status, poor	25.1%	25.0%	20.6%
Educational attainment, lower	17.7%	29.7%	60.3%
Living arrangement, living alone	13.6%	11.3%	23.0%
Marital status, not marriedª	47.8%	23.0%	32.8%
Current work status, not working	16.7%	16.2%	66.2%
Number of comorbidities^b^, one or more	5.0%	25.6%	65.3%
K6^c^ (/24), mean	5.3	4.4	4.2
Impairment of IADL	-	-	11.9%

Abbreviations: SD, standard deviation; BM, balanced meal; IADL, instrumental activities of daily living

ª Not married, divorced, separated, widowed, or never married

^b^ Hypertension, diabetes, stroke, heart disease, and cancer

^c^ 6-item Kessler psychological distress scale

Overall, there were no major differences in the percentage of individuals with poor SFS among the three age groups. However, educational attainment was found to be lower in older adults (60.3%) compared to young (17.7%) and middle-aged adults (29.7%). Additionally, the percentage of participants with unhealthy dietary habits (i.e., a low frequency of balanced meal consumption and skipping breakfast) tended to be higher in the young and middle-aged adult groups.

Table 2 shows the results of the Poisson regression models, which were used to examine the interactive effects of educational attainment and SFS on skipping breakfast, while adjusting for covariates, among the three age groups. The regression models revealed that lower educational attainment among young and middle-aged adults was significantly associated with a higher prevalence rate (PR) for skipping breakfast (PR: 1.20; 95% CI: 1.04, 1.39; p = 0.014; and PR: 1.44; 95% CI: 1.25, 1.66; p < 0.001, respectively) compared to higher educational attainment, but not poor SFS and no significant interactive effect was observed. Among older adults, lower educational attainment and poor SFS were significantly associated with a higher PR for skipping breakfast (PR: 1.29; 95% CI: 1.04, 1.60; *p* = 0.020; and PR: 1.61; 95% CI: 1.18, 2.19; *p* = 0.002, respectively) without any interaction.

**Table 2:** Prevalence ratios (PRs) for skipping breakfast by socioeconomic status^a^

	Reference	Category	Main effect			Interaction		
			PR	95% CI	*p*	PR	95% CI	*p*
**Young adults (n = 2,179)**								
Socioeconomic status								
EA	Higher	Lower	**1.21**	**1.09–1.36**	**0.001**	**1.20**	**1.04–1.39**	**0.014**
SFS	Moderate	Poor	**1.15**	**1.03–1.27**	**0.009**	1.14	1.01–1.29	0.035
EA × SFS^b^								0.796
**Middle-aged adults (n = 2,992)**								
Socioeconomic status								
EA	Higher	Lower	**1.42**	**1.27–1.60**	**< 0.001**	**1.44**	**1.25–1.66**	**< 0.001**
SFS	Moderate	Poor	1.10	0.97–1.25	0.123	1.12	0.95–1.33	0.182
EA × SFS^b^								0.743
**Older adults (n = 3,184)**								
Socioeconomic status								
EA	Higher	Lower	1.02	1.00–1.05	0.095	**1.29**	**1.04–1.60**	**0.020**
SFS	Moderate	Poor	1.04	1.00–1.07	0.032	**1.61**	**1.18–2.19**	**0.002**
EA × SFS^b^								0.042

Abbreviations: PR, prevalence ratio; CI, confidence interval; EA, educational attainment; SFS, subjective financial status

**Bold** values denote significant at α = 0.025 (0.05/2) by Bonferroni’s correction adjustment

ª Poisson regression model with robust variance. Dependent variable: skipping breakfast; Independent variable: socioeconomic status;

Covariates: sex, age, living arrangement, marital status, current work status, number of comorbidities, K6 and IADL (only for older adults)

^b^ Overall test of interaction

**Fig. 1:**
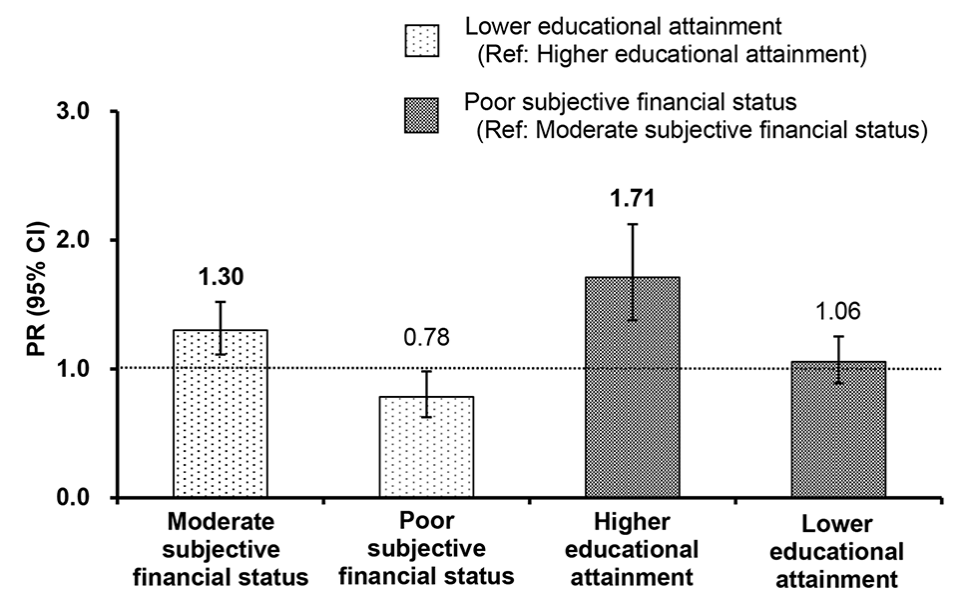
Results of simple main effect tests stratified by subjective financial status and educational attainment Leftmost bar: The result of the analysis was limited to those with moderate subjective financial status, indicating the PR of the low frequency of balanced meal consumption for lower education relative to higher education Second bar from left: The result of the analysis was limited to those with poor subjective financial status, indicating the PR of the low frequency of balanced meal consumption for lower education relative to higher education Third bar from left: The result of the analysis was limited to those with higher educational attainment, indicating the PR of the low frequency of balanced meal consumption for poor subjective financial status relative to moderate subjective financial status Rightmost bar: The result of the analysis was limited to those with lower educational attainment, indicating the PR of the low frequency of balanced meal consumption for poor subjective financial status relative to moderate subjective financial status Abbreviations: PR, prevalence ratio; CI, confidence interval **Bold** values denote significance at α = 0.0125 (0.05/4) by Bonferroni’s correction adjustment Each regression model was adjusted for sex, age, living arrangement, marital status, current work status, number of comorbidities, the 6-item Kessler Psychological Distress Scale score, and instrumental activities of daily living

Table 3 shows the results of the Poisson regression models examining the association between SES and low frequency of balanced meal consumption across the three age groups. Among young adults, a low frequency of balanced meal consumption was significantly associated with a higher PR for poor SFS compared to moderate SFS (PR: 1.10; 95% CI: 1.02, 1.19; *p* = 0.017). Middle-aged adults with lower educational attainment showed a significant association with a higher PR for low frequency of balanced meal consumption compared to those with higher educational attainment (PR: 1.17; 95% CI: 1.06, 1.28; *p* = 0.001). For older adults, both lower educational attainment (PR: 1.31; 95% CI: 1.12, 1.52; *p* = 0.001) and poor SFS (PR: 1.76; 95% CI: 1.43, 2.17; *p* < 0.001) were significantly associated with a higher PR for low frequency of balanced meal consumption, with a significant interaction (*p* < 0.001). Simple main effect tests, stratified by each factor (Fig. 1), revealed that among the moderate SFS group, a significantly higher PR for low frequency of balanced meal consumption was found in the lower educational attainment group (Ref: higher educational attainment; PR: 1.30; 95% CI: 1.11, 1.52; *p* = 0.001). Furthermore, among the higher educational attainment group, the poor SFS group was associated with significantly higher PRs for low frequency of balanced meal consumption (Ref: moredate SFS group; PR: 1.71; 95% CI: 1.38, 2.12; *p* < 0.001).

**Table 3:** Prevalence ratios (PRs) for a low frequency of balanced meal consumption by socioeconomic status^a^

	Reference	Category	Main effect			Interaction		
			PR	95% CI	*p*	PR	95% CI	*p*
**Young adults (n = 2,182)**								
Socioeconomic status								
EA	Higher	Lower	**1.12**	**1.04–1.21**	**0.004**	1.11	1.01–1.23	0.040
SFS	Moderate	Poor	**1.11**	**1.03–1.19**	**0.004**	**1.10**	**1.02–1.19**	**0.017**
EA × SFS^b^								0.792
**Middle-aged adults (n = 2,998)**								
Socioeconomic status								
EA	Higher	Lower	**1.14**	**1.95–1.23**	**0.001**	**1.17**	**1.06–1.28**	**0.001**
SFS	Moderate	Poor	1.01	0.93–1.10	0.760	1.05	0.94–1.16	0.382
EA × SFS^b^								0.285
**Older adults (n = 3,184)**								
Socioeconomic status								
EA	Higher	Lower	1.14	1.00–1.30	0.044	**1.31**	**1.12–1.52**	**0.001**
SFS	Moderate	Poor	**1.24**	**1.09–1.43**	**0.002**	**1.76**	**1.43–2.17**	**< 0.001**
EA × SFS^b^								**< 0.001**

Abbreviations: PR, prevalence ratio; CI, confidence interval; EA, educational attainment; SFS, subjective financial status

**Bold** values denote significant at α = 0.025 (0.05/2) by Bonferroni’s correction adjustment

ª Poisson regression model with robust variance. Dependent variable: low frequency of balanced meal consumption; Independent variable: socioeconomic status; Covariates: sex, age, living arrangement, marital status, current work status, number of comorbidities, K6, and IADL (only for older adults)

^b^ Overall test of interaction

Sensitivity analyses were performed using complete-case data of 8,464 participants (2,185 young adults aged 20–39 years; 3,008 middle-aged adults aged 40–64 years; 3,271 older adults aged 65 years or older; Table S1). The robustness of the findings was confirmed by comparing the results with the analysis outcomes of the complete-case data, excluding those with missing values.

## Discussion

This study aimed to investigate whether the association between education and dietary habits may be confounded by SFS and whether this effect varies across different age groups (generations), with a focus on skipping breakfast and low frequency of balanced meal consumption. The findings indicate that in older adults, there was an interactive effect of education and SFS on a low frequency of balanced meal, whereas, in young and middle-aged adults, there were only main effects of education and/or SFS on unhealthy diet. This finding suggests that the influence of each indicator differs among older adults, depending on their combination. Our results indicate that the influence of SES on dietary habits differed across generations, where older adults with poor SFS despite higher education and those with good SFS despite lower education were more likely to fall into unhealthy dietary habits. The findings suggest that it is important to consider different SES factors, including education and SFS, to understand their potential influence on dietary habits.

### SES and skipping breakfast

This study observed main effects of education and/or SFS on skipping breakfast all three age groups, without significant interaction, which is consistent with previous findings indicating a higher prevalence of skipping breakfast among those with poor SES [10, 28, 29]. The results further indicate that the association between lower educational levels and frequent breakfast skipping may result from inadequate knowledge and educational opportunities regarding healthy dietary habits, which are less accessible to individuals with lower educational levels [30, 31]. These findings are in line with research showing that poor nutrition knowledge may lead to skipping breakfast [32, 33], lending further support to the observed association between lower education and skipping breakfast.

Another possible explanation for the association between a lower education and frequent breakfast skipping is the level of health literacy, which is defined as the knowledge, willingness, and ability to obtain, understand, evaluate, and use health information [34]. Educational attainment is strongly linked to health literacy [35], and people with inadequate health literacy are less responsive to health education and less successful in managing chronic diseases [36]. Nutrition knowledge is an integral part of health literacy, and the use of media to obtain nutrition information and trust in nutrition information sources are associated with health literacy levels [37]. Therefore, people with low health literacy may not be able to obtain nutrition knowledge correctly, apply it meaningfully, or connect it to their habitual breakfast consumption [38]. These findings suggest the need for intervention activities to address health literacy issues and comprehensive promotion of healthy dietary behaviors. Considering that family and the environment, including the media, contribute to improving health literacy among young people [39, 40], our findings highlight the importance of implementing multifaceted interventions aimed at improving health literacy from an early age to reduce breakfast skipping.

Individuals with lower levels of education often have lower outcome expectations and fewer external cues motivating them to adopt health behaviors compared to those with higher education levels [41]. This may lead to less prioritization of the long-term health benefits of breakfast consumption. However, social interactions with family and friends may have a positive effect on promoting healthy behaviors among individuals with lower levels of education, and help them to better understand the direct health benefits of consuming breakfast [41]. Therefore, social influences, such as family and friends, could play an important role in reducing educational disparities in breakfast consumption.

This study did not find an interaction between educational attainment and SFS on skipping breakfast. Educational disparities in breakfast consumption were observed across all generations, suggesting that educational attainment, generally determined at a young age, strongly influences breakfast consumption throughout an individual’s lifetime. This finding is consistent with prior literature that has demonstrated an association between low education and skipping breakfast, even among older adults [42]. The habit of skipping breakfast is a reflection of irregular dietary habits, and this study measured the achievement of regular dietary habits among participants. Skipping breakfast can promote unhealthy dietary behaviors, such as overeating during subsequent meals and eating late at night, thereby contributing to poor health outcomes [6, 7, 9]. Educational disparities in breakfast consumption have been shown to contribute to social inequalities in health status. Therefore, educational interventions such as health education programs could be effective in enhancing nutrition knowledge and health literacy, which may contribute to the prevention of skipping breakfast, particularly among those with lower education and irregular dietary habits.

Poor SFS was also associated with skipping breakfast in older adults but not in young and middle-aged adults, suggesting that irrespective of education, older adults with poor financial status are more likely to skip breakfast. Low incomes place older adults at a greater risk of a nutritional deficiencies compared to other age groups [43], and older adults with poor SFS may be forced to skip breakfast. Therefore, policy-level interventions are needed to minimize the influence of economic disparity on breakfast skipping among older adults.

### SES and low frequency of balanced meal consumption

This study found differences in the association between SES and balanced meal consumption across the three generations. Specifically, there were main effects of SFS on balanced meal consumption in young adults, educational attainment in middle-aged adults, and an interactive effect of both in older adults. Therefore, our results highlight that the influence of education and SFS on balanced meal consumption varies across generations.

Previous research has suggested an association between SFS and balanced meal consumption [44], which has also been observed among older adults [45]. A systematic review of food costs and quality indicates that individuals with low incomes struggle to maintain healthy diets owing to the high costs of healthy foods, resulting in inequalities in diet quality [16]. According to the results of the National Health and Nutrition Survey in Japan, individuals with a household income of less than two million yen (approximately US $15,019) do not consume an adequately nutritionally balanced diet [24]. The fact that the association between low SFS and low frequency of balanced meal consumption was only observed in young adults, and not in middle-aged and older age groups may be due to increased health awareness among middle-aged and older adults and the relatively low number of middle-aged and older adults with household incomes below two million yen. Further research studies focusing on low-income groups may help clarify the association between low SFS and low frequency of balanced meal consumption among middle-aged and older adults.

Contrary to young adults, this study found a significant association between educational attainment and balanced meal consumption was observed only among middle-aged adults, suggesting that higher education equips middle-aged adults with the necessary knowledge to prioritize balanced meal consumption, irrespective of their financial status. Middle-aged adults with higher education are likely to prioritize balanced meal consumption, even when faced with economic challenges, whereas this is not the case for younger and older adults. This association between education and balanced meal consumption among middle-aged adults may also be attributed to their higher levels of health literacy and motivation that result from their higher levels of education, similar to the association found for skipping breakfast.

In addition, the study found that the interactive effect of education and SFS on balanced meal consumption was only significant in older adults. Specifically, significantly higher odds for a low frequency of balanced meal consumption were observed in those with lower education and moderate SFS and in those with higher education and lower SFS. Previous research has shown that not only do low-income older adults have lower balanced meal consumption [45], but also that education inequalities are associated with adherence to vegetable intake guidelines [46] and dietary intake [47] among older adults. The present study extends these findings and confirms the interaction of both education and SFS in older adults. Post-secondary or tertiary education is commonly viewed as an investment in an individual’s financial satisfaction [48], and not meeting these economic expectations may negatively impact the mental health of older adults, considering individuals’ natural tendency to compare their social standing to that of their immediate reference groups [49]. Mental health problems may lead to a loss of healthy dietary habits, including balanced meal consumption, among older adults with higher education and poor financial status. On the contrary, those with lower education and higher financial status may have a higher risk of low frequency of balanced meal consumption due to an unbalanced diet (e.g., a meat-based diet). The findings suggest that the influence of lower SES indicators (less educated or poor financial status) on balanced meal consumption may be stronger among older adults than among young and middle-aged adults, given the economic and socio-cultural context of the time [50]. Future studies should explore this mechanism in older adults.

The absence of a synergistic effect between lower education and poor financial status at risk factors for balanced meal consumption could be due to several reasons. One possible explanation is that individuals with lower education and financial status may be satisfied with their meals and perceive them as well-balanced, due to their limited understanding of health benefits and low motivation toward healthy eating for disease prevention [51]. However, it should be noted that the current discussions regarding the interactive effect of education and SFS on balanced meal consumption is based on speculation and should be examined in greater detail in future studies.

Nonetheless, the current study is significant in that it features one of the largest multi-generational sample sizes to analyze the association between SES and dietary habits and is the first study to indicate that education and SFS exert different influences on healthy dietary habits in different generations [52]. However, this study also has some limitations. First, our sample was composed of individuals living in a suburb who voluntarily participated in the mail survey, which could limit the generalizability of our results. Second, our cross-sectional design precluded us from drawing inferences about the likely causal relationships between SES and dietary habits. Third, our questionnaire regarding skipping breakfast did not consider the content of breakfast. Finally, given the inconsistent definition of skipping breakfast used in previous studies [53], caution should be exercised in interpreting the results for skipping breakfast. These limitations highlight the need for further research in these areas.

## Conclusions

This study reveals that the association between SES (education and SFS) and healthy dietary habits varies based on generational and SES indicators. We found that the effects of education and/or SFS were more pronounced among young and middle-aged adults, whereas older adults exhibited an interactive effect between the two indicators. These generational differences suggest that health policies aimed at promoting healthier dietary habits should consider the potential influence of different SES indicators, rather than just focusing on those with lower SES.

## Electronic supplementary material

Below is the link to the electronic supplementary material.

Supplementary Material 1

## Data Availability

The datasets generated and/or analyzed during the current study are not publicly available due to ethical requirements to maintain confidentiality, but are available from the corresponding author upon reasonable request.
