# Supplementary material for: Influence of education and subjective financial status on dietary habits among young, middle-aged, and older adults in Japan: a cross-sectional study
Source: BMC Public Health. 2023 Jun 26;23:1230. doi: 10.1186/s12889-023-16131-7 (PMC10291788; doi:10.1186/s12889-023-16131-7)
Supplement: Supplementary file 1 — Supplementary Material 1 [file 12889_2023_16131_MOESM1_ESM.docx]

**Supporting information**

Additional supporting information can be found in the online version of this article on the publisher’s website.

**Additional file 1: Supplementary Table 1**

Sociodemographic characteristics of participants by age group (complete case data)

**Supplementary Table 1**

Sociodemographic characteristics of participants by age group (complete case data)

|  | **Young adults**  **(n = 2,185)** | | **Middle-aged adults**  **(n = 3,008)** | | **Older adults**  **(n = 3,271)** | |
| --- | --- | --- | --- | --- | --- | --- |
|  | **n** | **Statistics** | **n** | **Statistics** | **n** | **Statistics** |
| Sex, male | 2,185 | 843 (38.6) | 3,008 | 1,336 (44.4) | 3,271 | 1,485 (45.4) |
| Age (years), mean (SD) | 2,185 | 31.2 (5.6) | 3,008 | 52.0 (7.1) | 3,271 | 75.0 (6.6) |
| Low frequency of BM consumption | 2,182 | 1,373 (62.9) | 2,998 | 1,452 (48.4) | 3,238 | 781 (24.1) |
| Skipping breakfast | 2,179 | 937 (43.0) | 2,992 | 846 (28.3) | 3,184 | 470 (14.8) |
| Subjective financial status, poor | 2,185 | 548 (25.1) | 3,008 | 752 (25.0) | 3,271 | 674 (20.6) |
| Educational attainment, lower | 2,185 | 387 (17.7) | 3,008 | 894 (29.7) | 3,271 | 1,973 (60.3) |
| Living arrangement, living alone | 2,170 | 295 (13.6) | 2,951 | 326 (11.0) | 3,103 | 651 (21.0) |
| Marital status, not marriedª | 2,181 | 1,042 (47.8) | 3,000 | 688 (22.9) | 3,232 | 1,054 (32.6) |
| Current work status, not working | 2,169 | 362 (16.7) | 2,953 | 476 (16.1) | 2,706 | 1,735 (64.1) |
| Number of comorbidities^b^, one or more | 2,113 | 106 (5.0) | 2,933 | 753 (25.7) | 3,192 | 2,086 (65.4) |
| K6^c^ (/24), mean | 2,172 | 5.3 (5.0) | 2,979 | 4.4 (4.3) | 3,047 | 4.1 (4.0) |
| Impairment of IADL | - | - | - | - | 3,221 | 381 (11.8) |

Abbreviations: SD, standard deviation; BM, balanced meals; IADL, instrumental activities of daily living.

ª Not married, divorced, separated, widowed, or never married.

^b^ Hypertension, diabetes, stroke, heart disease, and cancer.

^c^ 6-Item Kessler Psychological Distress Scale.
